# Flow diversion for cerebral aneurysms

**DOI:** 10.3171/2022.7.FOCVID2253

**Published:** 2022-10-01

**Authors:** Joseph A. Carnevale, Jacob L. Goldberg, Gary Kocharian, Andrew L. A. Garton, Alexander Ramos, Justin Schwarz, Srikanth Reddy Boddu, Y. Pierre Gobin, Jared Knopman

**Affiliations:** Department of Neurological Surgery, Weill Cornell Medicine/NewYork-Presbyterian Hospital, New York, New York

**Keywords:** cerebral aneurysms, flow diversion, ruptured aneurysm, giant aneurysm, posterior circulation aneurysm, vertebrobasilar aneurysms

## Abstract

The treatment of cerebral aneurysms includes open microsurgical options (e.g., clipping, trapping/bypass) and evolving endovascular techniques. Following the landmark trials that propelled endovascular treatment to the forefront, flow diversion has shown high aneurysm cure rates with minimal complications. Flow diversion stents are placed in the parent vessel, redirecting blood flow from the aneurysm, promoting reendothelization across the neck, and resulting in complete occlusion of the aneurysm. As a result, flow diversion has become increasingly used as the primary treatment for unruptured aneurysms; however, its applications are being pushed to new frontiers. Here, the authors present three cases showcasing the treatment of intracranial aneurysms with flow diversion.

The video can be found here: https://stream.cadmore.media/r10.3171/2022.7.FOCVID2253

## Transcript

Here, we present several cases showcasing the endovascular treatment of intracranial aneurysms with flow diversion.

### 0:27 Giant MCA Aneurysm Case Presentation.

First is a case that highlights flow diversion for a giant middle cerebral artery (MCA) aneurysm.

Here, a 25-year-old male was found to have a 3.7-cm right MCA aneurysm on workup for worsening headaches. Initially, CT/CTA showed a partially thrombosed 3.7-cm broad-based aneurysm arising from the mid- to distal M1 segment of the right MCA.

The DSA and 3D vessel reconstruction revealed the giant right distal MCA fusiform aneurysm, noting that components are partially thrombosed while patent components measure 3.5 cm wide × 2.5 cm tall. The superior and inferior divisions of the M2 segments maintain patency and continue beyond the base of the aneurysm.

### 1:14 Treatment of Giant MCA Aneurysm.

For treatment of this aneurysm, a triaxial system was utilized for stent placement while jailing a coiling microcatheter within the aneurysm.

Using roadmap technique and fluoroscopic guidance, the system was navigated into the right superior M2 in anticipation of stent deployment from this vessel to span across the aneurysm. This required a loop-the-loop maneuver within the aneurysm dome to come out the distal ostium into the distal MCA. Next, to assist with reducing this tortuosity and looping of the microcatheter system within the aneurysm, a Medtronic Solitaire 4 × 20–mm stent was partially deployed in the superior M2 division through the microcatheter to provide an anchor from which the tortuosity of the microcatheter could be reduced. The Solitaire stent was then removed from the microcatheter after successful reduction of the system.

Subsequently, a 3.25 × 35–mm Medtronic Pipeline Flex stent was then deployed from the right superior division of M2 to span the entirety of the fusiform aneurysm, with the proximal landing zone in the healthy right M1 segment. Numerous additional coils were then deployed, with intermittent fluoroscopy to ensure successful embolization.

Postembolization angiogram demonstrated a successfully embolized right MCA giant aneurysm. The dome was secured and there was appropriate contrast stagnation in dependent and base portions, consistent with successful early flow diverter stent embolization.

The patient awoke at his neurological baseline in the neurological ICU, discharged on postoperative day 1 with no complications, and plans for follow-up angiogram in 6 months.

### 2:49 Follow-Up of Giant MCA Aneurysm.

The 6-month follow-up DSA revealed complete embolization of the aneurysm, including the previously stagnating dependent and base portions. At this point, the ticagrelor was discontinued, and the patient was continued on aspirin with plans for a follow-up MRA in 1 year.

### 3:06 AICA Aneurysm Case Presentation.

Next is a case that highlights flow diversion for a ruptured posterior circulation aneurysm.

A 55-year-old female with a known fusiform right anterior inferior cerebellar artery (AICA) aneurysm presented with severe headache and was found to have Hunt-Hess 3, modified Fisher 3 subarachnoid hemorrhage in the right cerebellopontine and basal cisterns. A right frontal EVD was placed and the patient was admitted to the neuro ICU, where she was stabilized and our aneurysmal subarachnoid hemorrhage protocol was enacted.

Following hemodynamic stabilization and EVD placement, a CT/CTA head confirmed adequate placement of the EVD and stable subarachnoid blood. The CTA also revealed the culprit aneurysm located along the proximal right AICA. The patient was brought to the endovascular suite for definitive treatment of the ruptured aneurysm.

### 3:58 Treatment of AICA Aneurysm.

Diagnostic angiogram and 3D vessel reconstruction was conducted from the right vertebral artery. Along the right AICA there is 1 distinct aneurysm, fusiform in shape, measuring 6.2 × 4.8 mm in maximal diameter with a 5.2-mm neck.

At this point, the decision was made to proceed with flow diversion embolization of the aneurysm via the right radial artery. Using a 7-Fr radial sheath, the triaxial system was advanced into the right vertebral artery. With the intermediate catheter parked in the right V4 segment, the microcatheter was advanced over the microwire into the right AICA, distal to the aneurysm. A Medtronic Pipeline Flex 2.5 × 10–mm stent was advanced via the microcatheter. The stent markers were positioned across the aneurysm neck in the desired position and deployed with the distal end passing the base of the aneurysm into the distal AICA and the proximal end at the origin of AICA.

Postembolization angiogram confirmed stent patency, juxtaposition of the stent with the parent vessel, and coverage of the aneurysm with moderate stasis of contrast noted within the aneurysm. No in-stent thrombosis or distal emboli as well as normal antegrade flow is demonstrated in the basilar artery and distal branches of AICA following stent placement.

The patient was started on a cangrelor drip and returned to the neuro ICU at her neurological baseline. She was transitioned to oral aspirin and ticagrelor. Her hospitalization was otherwise uneventful, no vasospasm occurred, and EVD clamp trial successful. The patient was discharged home from the ICU on postoperative day 15 at her neurological baseline.

### 5:38 Follow-Up of AICA Aneurysm.

The 3-month follow-up DSA revealed complete embolization of the right AICA aneurysm. At this point, the ticagrelor was discontinued, and the patient was continued on aspirin with plans for a follow-up DSA in 1 year.

### 5:52 Vertebrobasilar Aneurysm Case Presentation.

Our final case demonstrates the use of coil embolization, vessel sacrifice, and flow diversion for an aneurysm of the vertebrobasilar junction.

A 64-year-old female with poorly controlled diabetes, hypertension, hyperlipidemia initially presented with 2 weeks of headache, nausea, vomiting, and 1 day of altered mental status.

Of note, serial imaging revealed a vertebrobasilar artery mycotic aneurysm associated with new multifocal small acute infarcts involving deep gray structures.

Once stabilized, the patient was taken to the neuroendovascular suite for DSA and definitive treatment of vertebrobasilar aneurysm.

### 6:30 Treatment of Vertebrobasilar Aneurysm.

Diagnostic angiogram and 3D vessel reconstruction revealed a laterally projecting aneurysm, measuring approximately 4.4 mm in maximal diameter at the left vertebrobasilar junction.

At this point, the decision was made to proceed with flow diversion embolization of the aneurysm via the right radial artery. Using a 6-Fr radial sheath, the triaxial system was advanced over the microwire into the left vertebral artery. The microcatheter was subsequently advanced beyond the aneurysm and into the basilar artery. A Pipeline Flex 4 × 14–mm stent was advanced via the microcatheter. The stent markers were positioned across the aneurysm neck in the desired position, well opposed to the parent vessel. There is significant stagnation of contrast in the aneurysm.

Next, a microcatheter was advanced over its wire to the distal right vertebral artery. Under fluoroscopic guidance, coils were deployed into the distal right vertebral artery with the flow-diverting stent as backboard. Following coil embolization, right vertebral artery angiogram reveals anterograde flow into the right posterior inferior cerebellar artery (PICA), no flow distal to the coil mass, and no continued filling of the aneurysm.

### 7:43 Follow-Up of Vertebrobasilar Aneurysm.

Following left vertebrobasilar aneurysm Pipeline stenting and right distal vertebral artery sacrifice with coil embolization for this mycotic aneurysm, the patient was loaded with aspirin and ticagrelor and transferred to neurological ICU for further postoperative care. Following a lengthy hospital course, the patient was discharged to acute rehab at her neurological baseline.

The 5-month follow-up CTA revealed complete embolization of the vertebrobasilar mycotic aneurysm. At this point, the ticagrelor was discontinued, and the patient was continued on aspirin 81 mg daily with plans for a follow-up DSA in 1 year.

### 8:18 Conclusions.

Overall, this Journal of Neurosurgery *Focus* video presentation highlights the wide-ranging capabilities of flow-diverting stents for the treatment of cerebral aneurysms. As shown here, flow diversion has become increasingly used as the primary treatment for unruptured intracranial aneurysms; however, its applications are being pushed to new clinical, anatomical, and morphological frontiers, further advancing the endovascular treatment and cure of cerebral aneurysms.

### 8:50 References^1–6^

## Acknowledgments

Special thank you to Donald Elivert, voice actor.

## Disclosures

Dr. Gobin: CEO and medical director of Serenity Medical, a medical device company developing a stent for treating venous sinus stenosis.

## Author Contributions

Primary surgeon: Boddu, Knopman. Assistant surgeon: Carnevale, Goldberg, Kocharian, Ramos, Gobin. Editing and drafting the video and abstract: Carnevale, Goldberg, Kocharian, Garton, Boddu, Knopman. Critically revising the work: Carnevale, Goldberg, Kocharian, Garton, Ramos, Boddu, Gobin, Knopman. Reviewed submitted version of the work: all authors. Approved the final version of the work on behalf of all authors: Carnevale. Supervision: Carnevale, Boddu, Gobin, Knopman.
